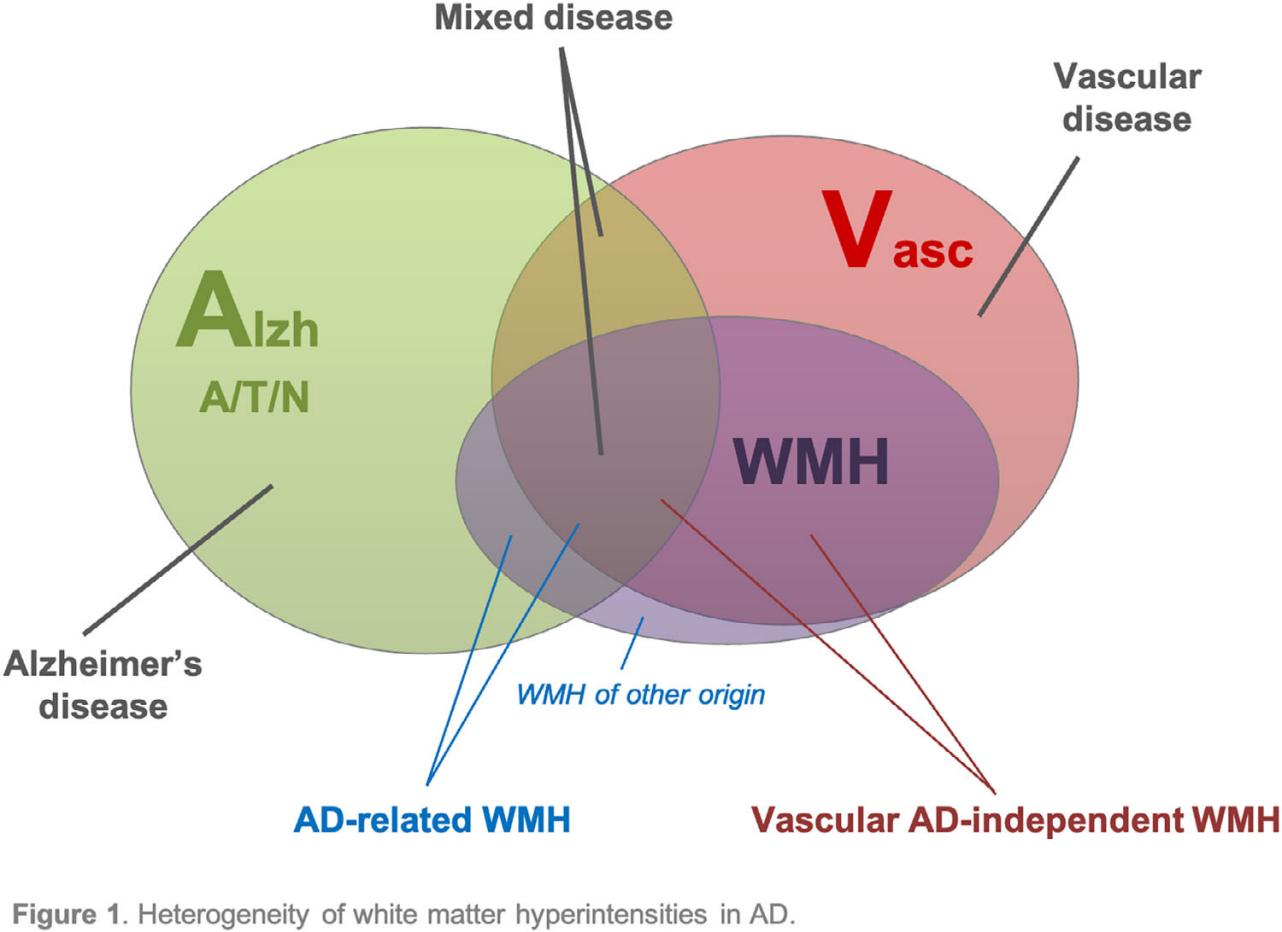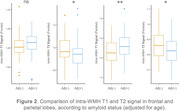# All WMH are not of purely vascular origin: existence of AD‐related WMH

**DOI:** 10.1002/alz.092168

**Published:** 2025-01-09

**Authors:** Antoine Garnier‐Crussard, Gael Chételat

**Affiliations:** ^1^ Clinical and Research Memory Center of Lyon, Lyon Institute For Ageing, Hospices Civils de Lyon, Lyon France; ^2^ Normandie Univ, UNICAEN, INSERM, U1237, PhIND "Physiopathology and Imaging of Neurological Disorders", Institut Blood and Brain @ Caen‐Normandie, Cyceron, Caen France; ^3^ Normandie Univ, UNICAEN, INSERM, U1237, PhIND "Physiopathology and Imaging of Neurological Disorders", NeuroPresage Team, GIP Cyceron, Caen France

## Abstract

White matter hyperintensities (WMH) are highly prevalent in aging and Alzheimer’s disease (AD) and typically attributed to vascular damage and cerebral small vessel disease. Yet, several lines of evidence from the literature emphasize the heterogeneity in the mechanisms leading to WMH, notably in AD, suggesting that WMH may be partly attributable to AD.

Thus, firstly, neuropathological studies demonstrate heterogeneity in WMH histology, with indications of a link between tau pathology, Wallerian degeneration, and WMH severity. Secondly, in AD, WMH are an early event, partly independent of vascular risk factors, suggesting a specific AD‐related pathway leading to WMH. Thirdly, WMH topography in AD differs from age‐related WMH, globally mirroring patterns of neurodegeneration. Fourthly, genetic studies and biomarker analyses further support the association between AD and WMH. Potential mechanisms for AD‐related WMH are Wallerian degeneration due to proteinopathies (tau and amyloid) and neuroinflammation – in addition to vascular mechanisms, including the contribution of cerebral amyloid angiopathy.

Building upon this evidence, we propose an alternative hypothesis challenging the exclusive vascular origin of WMH. This alternative hypothesis posits that, beyond purely vascular AD‐independent WMH, a subset of WMH, termed AD‐related WMH, is linked to AD pathology (see Figure 1).

To test the hypothesis of distinct etiologies for WMH in AD (vascular versus AD‐related), we extracted the signal within the WMH from different neuroimaging modalities (T1 and T2 MRI, AV45 and FDG PET) in 142 participants (IMAP cohort), and i) performed cluster analyses to reveal distinct clusters representing WMH with varied properties; and ii) compared clinical groups (cognitively unimpaired/impaired) or amyloid status (positive/negative) to highlight AD‐related differences in WMH signals. Our findings offer initial support for our hypothesis; they show for instance higher T1 and lower T2 intra‐WMH signal in the frontal and parietal lobe in amyloid positive compared to amyloid negative participants (Figure 2).

In conclusion, we advocate for a nuanced perspective of WMH etiology in AD, going beyond traditional vascular origins and acknowledging the potential contributions of AD‐related mechanisms. A comprehensive interpretation of WMH will undoubtedly advance our understanding of AD pathology and guide more effective diagnostic and therapeutic approaches.